# Highly regional population structure of *Spondyliosoma cantharus* depicted by nuclear and mitochondrial DNA data

**DOI:** 10.1038/s41598-020-61050-x

**Published:** 2020-03-04

**Authors:** Ana Neves, Ana Rita Vieira, Vera Sequeira, Rafaela Barros Paiva, Leonel Serrano Gordo, Octávio S. Paulo

**Affiliations:** 1 Departamento de Biologia Animal, Faculdade de Ciências, Universidade de Lisboa, Campo Grande, 1749-016 Lisboa, Portugal; 2 MARE – Marine and Environmental Sciences Centre, Faculdade de Ciências, Universidade de Lisboa, Campo Grande, 1749-016 Lisboa, Portugal; 3 cE3c - Centre for Ecology, Evolution and Environmental Changes, Faculdade de Ciências, Universidade de Lisboa, Lisboa, Portugal

**Keywords:** Evolutionary ecology, Population dynamics

## Abstract

Resolution of population structure represents an effective way to define biological stocks and inform efficient fisheries management. In the present study, the phylogeography of the protogynous sparid *Spondyliosoma cantharus*, in the East Atlantic and Mediterranean Sea, was investigated with nuclear (S7) and mitochondrial (cytochrome *b*) DNA markers. Significant divergence of four regional genetic groups was observed: North Eastern Atlantic, Mediterranean Sea, Western African Transition (Cape Verde) and Gulf of Guinea (Angola). The two southern populations (Cape Verde and Angola) each comprised reciprocally monophyletic mtDNA lineages, revealed low levels of diversity in Cape Verde and high diversity for Angola despite being represented by only 14 individuals. A complete divergence between North Atlantic and Mediterranean populations was depicted by the mitochondrial marker, but a highly shared nuclear haplotype revealed an incomplete lineage sorting between these regions. Bayesian skyline plots and associated statistics revealed different dynamics among the four regions. Cape Verde showed no expansion and the expansion time estimated for Angola was much older than for the other regions. Mediterranean region seems to have experienced an early population growth but has remained with a stable population size for the last 30000 years while the North Atlantic population has been steadily growing. The lack of genetic structuring within these regions should not be taken as evidence of demographic panmixia in light of potential resolution thresholds and previous evidence of intra-regional phenotypic heterogeneity.

## Introduction

An essential part of sustainable management of fisheries is the identification of biological fish stocks, as it is the unit at which population assessments should be undertaken and management measures are applied^1,2^. Determining the spatial patterns of demographic independence and mixing among post-juvenile fish populations is critical for sustainability of fisheries^3^. Molecular genetic methods have been used for a long time to infer stock structure in fishes, as they proved to be efficient tools at identifying demographically independent units for fisheries management^4^. Overfishing such units can lead to a collapse of the fishery since a recovery of population sizes from migration is unlikely to occur and with the loss of a genetic stock, a species also loses the individuals that are adapted to a particular habitat through evolution, i.e. local adaptation^5^.

Sparids are highly important fisheries resources, with numerous species being commercially exploited. One of these species is the black seabream, *Spondyliosoma cantharus*, that occurs over a wide range of the eastern Atlantic, from Scandinavia to Namibia, around Madeira, Cape Verde and the Canary Islands. It is also common in the Mediterranean Sea and the western Black Sea^6^. Landings have reached almost 10 000 t per year in the last decade, with a major input from the north and central Eastern Atlantic, showing an increasing trend since 1980^7^. Life history characteristics of *S. cantharus* make it particularly vulnerable to local over-exploitation since it is a protogynous hermaphrodite, slow-growing, long-lived^8^, showing habitat specificity during the spawning season, with spawning aggregations and male nest guarding behaviours^9,10^.

Despite the wide geographical distribution of *S. cantharus*, little information on stock structure is available for the species. Only two studies on body morphology^11^ and otolith shape and isotopes ratios^12^ have analysed the population structure of the species, revealing clear distinct morphotypes for the Canary Islands and Angola and evident phenotypic differences among several of the European areas analysed. The use of genetic approaches along with phenotypic ones is advised in order to create an interdisciplinary perspective and increase the probability of correct stock identification^13^. In this study the phylogeography and population structure of *S. cantharus* was explored across the East Atlantic and Mediterranean Sea using both mitochondrial (cytochrome *b*) and nuclear (S7) DNA, in order to identify putative stocks and compare the results to those found for the phenotypic traits.

## Results

A total of 263 cyt*b* sequences were obtained, aligned, and trimmed to 663 bp, with 63 distinct haplotypes identified (GenBank accession nos. MH545766 – MH545828) showing 99 polymorphic sites yielding 81 transitions and 26 transversions. For S7, 366 sequences (corresponding to 183 individuals) with 483 bp were analysed and 85 haplotypes were identified (GenBank accession nos. MH545829 – MH545911), with 90 polymorphic sites representing 44 transitions, 34 transversions and 16 sites showed insertion/deletion variation in a total of 7 indel events with an average indel length event of 2.857.

### Population structure

**Figure 1:**
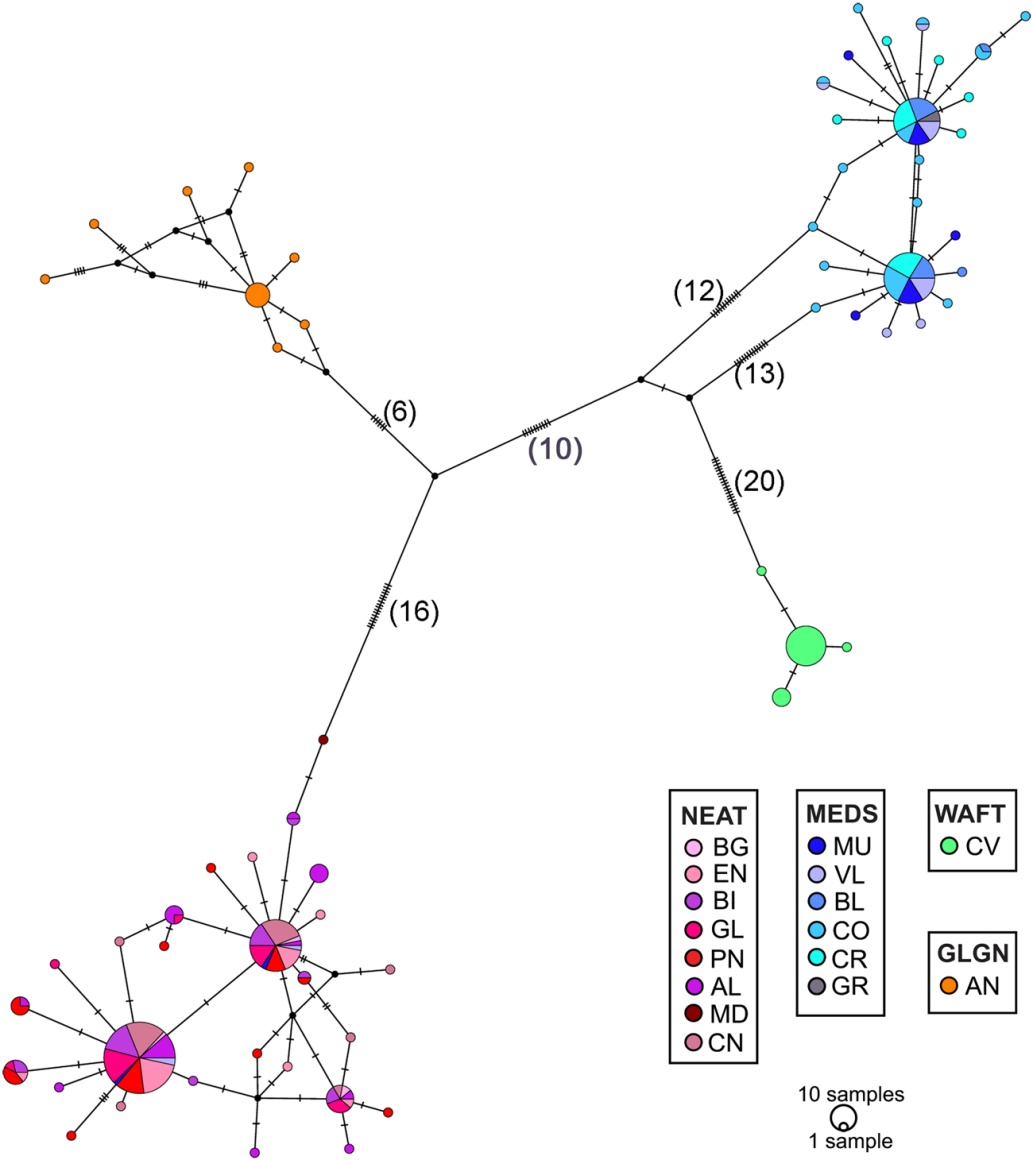
Parsimony network for *Spondyliosoma cantharus* calculated with mtDNA cytochrome *b* sequences (663 bp). The area of the circles is proportional to each haplotype frequency. Dashes represent mutations and the number of mutations between group regions are given between brackets. Colours refer to the population in which haplotypes were found. Acronyms for populations as in sampling section.

**Figure 2:**
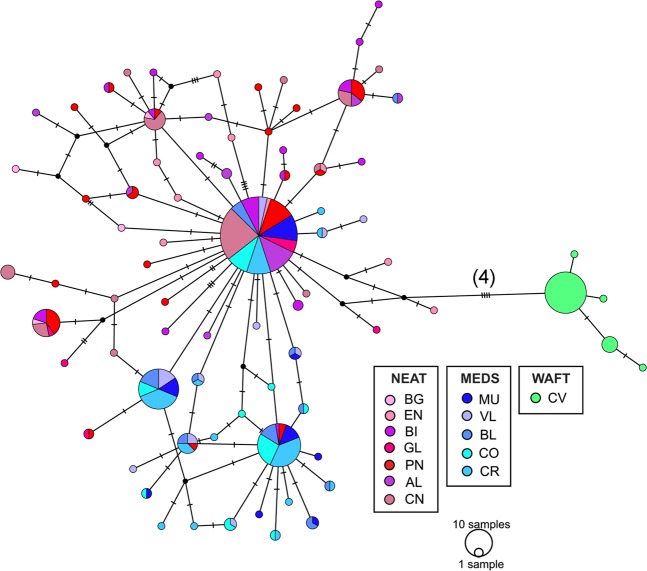
Parsimony network for *Spondyliosoma cantharus* calculated with nDNA first intron S7 sequences (483 bp). The area of the circles is proportional to each haplotype frequency. Dashes represent mutations and the number of mutations between Cape Verde and the remaining populations is given between brackets. Colours refer to the population in which haplotypes were found. Acronyms for populations as in sampling section.

The haplotype network for the cyt*b* of *S. cantharus* revealed four distinct groups (Fig. 1). The first group included mainly sequences from the North Eastern Atlantic (NEAT) areas (BG, EN, BI, GL, PN, AL and CN), with one haplotype shared among 61 individuals out of 263 and including three sampled in the Mediterranean Sea (MEDS), one from MU and two from VL. A second group, represented by MEDS sequences, showed two main haplotypes shared by 31 and 26 individuals from all the MEDS sampled areas. The two other groups were composed exclusively by private haplotypes, 4 for Cape Verde (West African Transition region, WAFT) and 8 for Angola (Gulf of Guinea region, GLGN). A similar pattern was present for the S7 haplotype network (Fig. 2), although not so distinct, since one haplotype occurred 110 times and was shared by all sampled areas from the NEAT and MEDS. However, only three more haplotypes were shared between NEAT and MEDS areas (Fig. 2, Supplementary Table S1) showing a distinct separation of the haplotypes occurring in NEAT and MEDS individuals. The Cape Verde samples showed, again, only private haplotypes. None of the DNA samples from AN could be amplified for the S7 region and therefore AN was excluded from S7 results.

A significant population structuring for both cyt*b* (F_ST_ = 0.928, p < 0.000) and the S7 (F_ST_ = 0.618, p < 0.000) was defined. Pairwise F_ST_ and Φ_ST_ values for the populations within each region (Supplementary Table S2), showed no structure among the populations with all non-significant values. Conversely, a significant genetic structure, with high F_ST_ and Φ_ST_ values between all regions, was estimated for both markers (Table 1). AMOVA indicated > 92% (cytB) and ~60% (S7) of variance to be due to differences among regions (Table 2).

**Table 1:** Gene flow among regions for *Spondyliosoma cantharus* represented by F_ST_ (below diagonal) and Φ_ST_ (above diagonal).

	cyt*b*				S7		
	NEAT	MEDS	WAFT	GLGN	NEAT	MEDS	WAFT
NEAT		**32.08**	**46.81**	**27.16**		**0.045**	**0.323**
MEDS	**0.914**		**35.31**	**30.31**	**0.0505**		**0.320**
WAFT	**0.970**	**0.895**		**41.98**	**0.293**	**0.288**	
GLGN	**0.940**	**0.861**	**0.969**				

Significant values of p < 0.05 assessed by permutation test with 1 × 10^5^ replicates are shown in bold. NEAT – North Eastern Atlantic; MEDS – Mediterranean Sea; WAFT – West African Transition; GLGN – Gulf of Guinea.

**Table 2:** Hierarchical analyses of molecular variance (AMOVA) for cyt*b* and S7 sequences of *Spondyliosoma cantharus*.

	source of variation	df	variance components	percentage of variation	Fixation index
**cyt*b*** 4 groups: CV vs AN vs NEAT vs MEDS	Among groups	3	**16.20**	92.44	F_ST_ 0.928
	Among populations within groups	10	**0.06**	0.36	
	Within populations	249	**1.26**	7.19	
**S7** 3 groups: NEAT vs MEDS vs CV	Among groups	2	**1.61**	61.51	F_ST_ 0.615
	Among populations within groups	10	**0.19**	0.7.30	
	Within populations	353	**0.81**	31.19	

Probability values were obtained after a permutation test with 1 × 10^5^ replicates and significance p < 0.05 level are indicated in bold. AN – Angola; CV – Cape Verde; NEAT – North Eastern Atlantic; MEDS – Mediterranean Sea.

Mantel test showed significant correlations between genetic and geographical distances for cyt*b* (r = 0.50, p < 0.001) but not for S7 (r = 0.51, p = 0.083). However, when the mantel test was carried out for populations within each region, isolation by distance was not found for either cyt*b* (r = 0.06, p = 0.370 and r = 0.14, p = 0.298 for NEAT and MEDS, respectively) or S7 (r = 0.56, p = 0.098 and r = −0.23, p = 0.623, NEAT and MEDS, respectively).

### Diversity analysis

**Figure 3:**
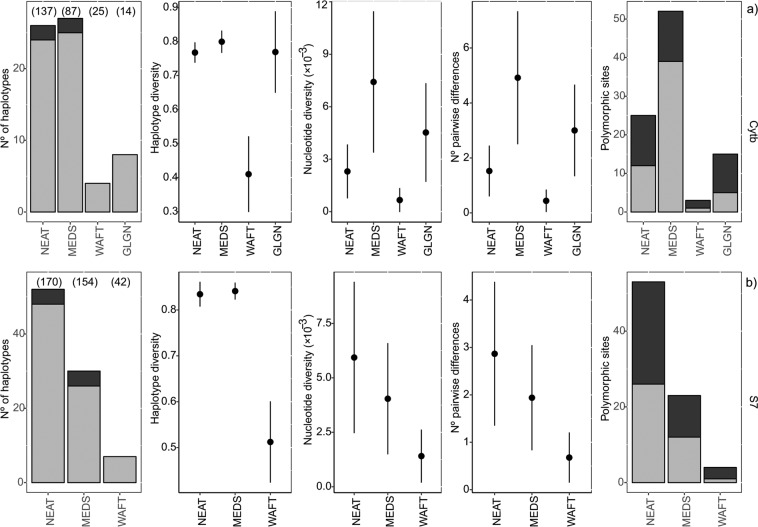
Standard diversity measures by regions for *Spondyliosoma cantharus* (**a**) cyt*b* and (**b**) S7. For each region, the number of haplotypes, haplotype diversity, nucleotide diversity, the mean number of pairwise differences and the number of polymorphic sites are shown. Number of haplotypes and polymorphic sites are divided in private/informative (grey) and shared/non informative (black). Vertical bars denote standard deviations. Number of individuals analysed are given in parenthesis above each region bar of the number of haplotypes graphic. NEAT – North Eastern Atlantic; MEDS – Mediterranean Sea; WAFT – West African Transition; GLGN – Gulf of Guinea.

A large proportion of singleton haplotypes were obtained for cyt*b* (73%) and S7 (62%). The WAFT region showed the lower values for all the diversity indices in both DNA markers (Fig. 3). The GLGN region showed high values for all indices estimated with cyt*b* marker, despite the low number of individuals sampled in this region (Fig. 3a). For cyt*b* the MEDS region showed the higher diversity indices. However, values for this region were inflated due to only five individuals of MU and VL populations that had shared haplotypes with the NEAT region (Supplementary Table S1). For S7 marker MEDS region showed slightly higher haplotype diversity, while NEAT showed higher values for the rest of the indices (Fig. 3b).

### Demographic analysis

Neutrality results suggest demographic expansion for NEAT and MEDS regions with significant negative values estimated (Table 3). For WAFT region the estimated values were negative but not significant (Table 3). For GLGN region only R_2_ test yield significant value for population growth, but given the small sample size of this region, the R_2_ test is expected to give the more reliable results^14^.

**Table 3:** Demographic parameters of *Spondyliosoma cantharus* based on cyt*b* and S7.

	NEAT	MEDS	WAFT	GLGN
	**neutrality tests**			
Tajima’s D	**−1.912**	**−1.694**	−1.108	−1.484
Fs	**−22.014**	**−8.188**	−1.653	−1.936
R_2_	**0.0299**	0.0448	0.1018	**0.0826**
	**mismatch distribution**			
	demographic expansion			
τ	1.516 (0.707–2.879)		0.5 (0–1.189)	9.2 (0.133–80)
t (ky)	143 (64–454)		71 (0–170)	1318 (19–11463)
SSD	0.0013	**0.0011**	0.0074	0.0199
Hri	0.0413	**0.0888**	0.1675	0.0494
	spatial expansion			
τ	0.980 (0.543–2.763)		0.526 (0.099–1.534)	6.797 (0.187–97.582)
t (ky)	140 (78–396)		75 (14–220)	974 (27–13982)
SSD	0.0013	**0.1073**	0.0073	0.0181
Hri	0.0413	**0.0888**	0.1675	0.0494
	**BEAST Bayesian Skyline**			
*t*_MRCA_ (ky)	225 (67–425)	160 (56–309)	81 (4–197)	713 (325–1147)

Neutrality tests: Fs (Fu’s), D (Tajima’s) and R2 test. Mismatch distributions: τ (age of expansion in units of mutational time) t (time in thousand years), SSD (sum of square deviation) and Hri (Harpending’s Raggedness index). Bayesian Skyline: tMRCA (time to most recent common ancestor in thousand years). Significant values of probability assessed by a permutation test with 1 × 10^5^ replicates are shown in bold and 95% CI are given in parenthesis. NEAT – North Eastern Atlantic; MEDS – Mediterranean Sea; WAFT – West African Transition; GLGN – Gulf of Guinea.

**Figure 4:**
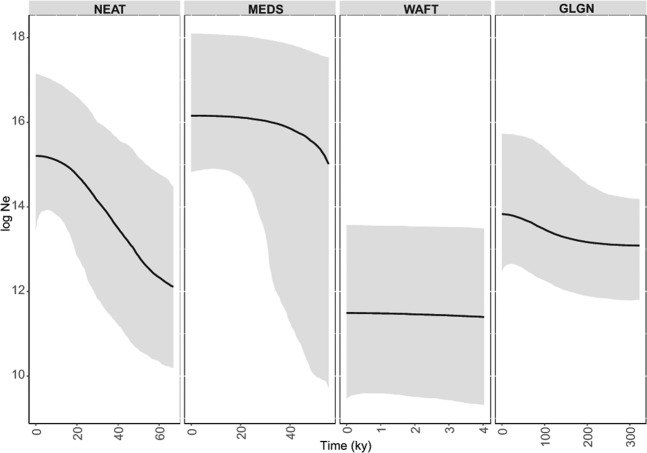
Bayesian skyline plot showing changes in effective population size through time (thousand years before present, ky) for *Spondyliosoma cantharus* for each region. The black line represents the median posterior estimate of the effective population size. The grey area shows the 95% highest posterior density limits. NEAT – North Eastern Atlantic; MEDS – Mediterranean Sea; WAFT – West African Transition; GLGN – Gulf of Guinea.

The mismatch distributions calculated for the different regions did not differ statistically from those expected for populations experiencing a demographic expansion and a spatial expansion, with exception of MEDS (Table 3, Supplementary Fig. S1). The average time estimated for demographic and spatial expansion showed similar values, reflecting an expansion period during the Pleistocene for GLGN region, while the other regions showed an expansion during the last glacial period. However, these values should be considered with caution due to the wide confidence intervals found. The tMRCA estimates were similar to the expansion estimates from mismatch analyses (Table 3). The Bayesian skyline plot showed no signatures of expansion for the WAFT region, with an effective population size much smaller than the other regions, while NEAT region has been steadily growing (Fig. 4). MEDS region seems to have experienced a population growth but has remained with a stable effective population size for the last 30 000 years (Fig. 4). The GLGN region showed an earlier expansion time and a slight increasing population trend with a peak about 100 000 years ago (Fig. 4).

## Discussion

In the present study the genetic structure of *S. cantharus* was analysed with nuclear and mitochondrial markers in 14 sampling areas along the geographical distribution of the species. The results revealed high regional population structure, with four clear groups in the analysed area: NEAT, MEDS, WAFT (represented by Cape Verde) and GLGN (represented by Angola). All the analyses clearly disclosed genetic structure among these regions, with each of the southern populations (GLGN and WAFT) comprising reciprocally monophyletic endemic clades and evidence of limited migration between NEAT and MEDS.

The Atlantic Ocean and the Mediterranean Sea are connected by the Strait of Gibraltar since the end of the Messinian Salinity Crisis (some 5.33 million years ago)^15^. The Strait of Gibraltar with 12.9 km wide and 286 m deep can represent a phylogeographical break, but no obvious relationship has been established between species dispersal ability or life history, and the observed patterns of genetic isolation between Atlantic and Mediterranean populations^15^. In the Sparidae family, divergent patterns have been observed for species with similar biology. A clear differentiation from Atlantic and Mediterranean populations have been found for species such as *Lithognathus mormyrus*, *Dentex dentex*^16^, *Diplodus puntazzo*^17^ but not for others like *Pagrus pagrus*^15,18^, *Pagellus bogaraveo*^16^, *P. erythrinus*^19^, *Diplodus sargus*^17,20^. For *S. cantharus*^16^, found different patterns when using mitochondrial DNA, where clear differentiation between Atlantic and Mediterranean populations was detected, while no differentiation was found for allozymes data. In the present study the cyt*b* marker evidenced a clear separation from NEAT and MEDS regions, with only two shared haplotypes that occurred in all NEAT populations and the two nearest MEDS populations, MU and VL. This points for a secondary contact with unidirectional gene flow from the Atlantic to the Mediterranean. The nuclear allele sharing could reflect a combination of nuclear introgression and incomplete lineage sorting. In fact, even if mutation rates were similar, mitochondrial DNA is expected to have lineage sorting four times faster than the nuclear loci, since the former have a single copy per individual and is inherited uniparentally and thus has a smaller genetically effective population size^21^. For protogynous fishes such as *S. cantharus*, this sorting difference may be somewhat smaller since all breeders contribute to the mitochondrial gene pool of the species. The mtDNA genomic compartment in this species can be ½ and not ¼ as for most species, because the same individual will mature as female and change sex along its life and consequently, “all” individuals will function as females first, transmitting a copy of mtDNA into the population.

*S. cantharus* populations within each region showed no population structure, with very low pairwise F_ST_ and Φ_ST_ values for both DNA markers, and lack of IBD found among samples collected within NEAT and MEDS regions. Within each region shared mitochondrial and nuclear haplotypes were present in all populations and nucleotide diversity showed no defined geographical trend with similar values for most populations. The high number of singletons found is also common among marine fishes, where the usual haplotype distribution for large fish populations is a small number in medium to high frequencies, with most occurring in low frequencies or in single copy^22^.

Despite efforts to gather samples from multiple areas within the Gulf of Guinea only samples from one population, Angola, could be collected. Cyt*b* results showed haplotypic diversity values similar to the NEAT and MEDS regions and higher nucleotide diversity despite the low number of individuals analysed. Expansion time for GLGN region dates from late Pleistocene, and these populations are likely to have maintained large number of individuals during glacial cycles since this area can function as tropical refugia where the mild conditions^23^ allowed to maintain a high number of breeders, justifying the high diversity found in the small number of individuals analysed. No data for the S7 could be obtained, since this DNA marker could not be amplified for the samples in this population. Nuclear DNA experience lower mutation rates than mitochondrial DNA^24^ and thus slower sorting among population is expected to occur, as discussed above for NEAT and MEDS regions. Unfortunately, the absence of nuclear DNA information for the GLGN region preclude to explore the occurrence of shared haplotypes with the northern regions, as described for other species such as *Pomatomus saltatrix*^25^.

The diversity indices found for both DNA markers in WAFT population were much lower than those estimated for the other areas. The much recent time of expansion estimated for this region can justify these low values since the population might have still had no time to highly increase diversity, persisting a single strong founder recolonization effect^26^. This region is strongly influenced by large-scale oceanic circulation and dominant currents which are likely to promote a physical barrier to larval drifting across the archipelago^27^ serving to restrict gene flow. The fact that fishing has been increasing in this region since 2005^28^ can also contribute to the decline of the genetic pool available in the archipelago, which can eventually lead to genetic drift in small populations^29^ reducing the potential of the population to adapt to rapid environmental changes.

The population expansion time in the NEAT and MEDS regions, as estimated by BSP, dates from the Last Glacial Period (LGP). These values must be looked with caution, since no molecular clock calibration is available for the species and the substitution rates inferred at population levels are much higher than those for species level^30–32^ holding a more recent time since expansion than that estimated from BSP and mismatch distribution. Nevertheless, it is likely that during the climatic oscillations that occurred over the LGP, the species had refugia in two different areas, one in the Mediterranean and one in the southern North Eastern Atlantic and that these populations have been evolving separately since then. Refugia in North Eastern Atlantic is probable to have occurred near south Iberian Peninsula. Although no latitudinal cline in allelic richness is present, populations from Portuguese coast showed slightly higher values.

The relatively long pelagic larval phase duration (PLD) reported for *S. cantharus*^33^ could also influence the low degree of genetic differentiation found along the European coasts, as the correlation between the PLD and F_ST_ estimates has been found to be very strong in recent modelling approaches^34^. However, phenotypic information available on the species points for segregation among NEAT populations, with clear morphological features of body and otoliths shape varying across the sampled areas^11,12^. The lack of within region genetic structure, with the low values of F_ST_ and Φ_ST_, can be due to just enough gene flow existing to homogenize neutral markers despite the limited exchange of individuals on average among sites. In fact, despite the proved value of neutral genetic markers in elucidating patterns of genetic connectivity among wild populations, they might not be appropriate or sensitive enough to detect shallow genetic structure or to provide information about adaptive genetic variation^35^. The values of divergence for quantitative trait loci between populations are expected to be much higher than those obtained with neutral markers^35^. Markers with high mutational rates (STRs) or a genome wide analysis (SNPs) or even candidate genes for environmental responses/morphological traits could provide a deeper insight of species stock structure (see^36^ and references therein). Several authors have already detected, for other species, no population structure with molecular markers but differentiation with phenotypical traits (eg^2,37,38^.), pointing to the importance from a fisheries management point of view that several methodologies should be gathered to establishing an effective knowledge of the species population structure.

## Conclusion

The obtained results reveal a large scale structure for *S. cantharus* populations. The Cape Verde and Angola regions revealed two distinct clades and the Northeast Atlantic and the Mediterranean populations showed a clear separation but with incomplete lineage sorting from a common ancestor revealed by the nuclear marker. Similar diversity was estimated for all populations analysed with exception of Cape Verde. The shallow diversity, low Ne, and seemingly isolated nature may compromise the resilience of the Cape Verde population to increasing fishing pressure and environmental changes and this should be taken in account and considered for future studies in the area.

Despite the evidence from the available phenotypic traits data of significant differences within NEAT populations, genetic data showed no differentiation among populations for both NEAT and MEDS regions. This indicates that gene flow exists among locations although with some restriction as suggested by some differentiation in derived, more recent mutations that account for morphological differentiation. The markers used in this study are good for analysing event at an evolutionary timescale but less powerful for detecting events occurring at an ecological timescale. The establishment of unequivocal stock structure of the species will benefit with the use of markers with high mutation rates. The knowledge on stock structure of exploited species is essential in a world where the rising demand for fish protein and consequent increase in fishing pressure combined with global warming boosts the challenges faced by these species.

## Material and Methods

### Sampling

**Figure 5:**
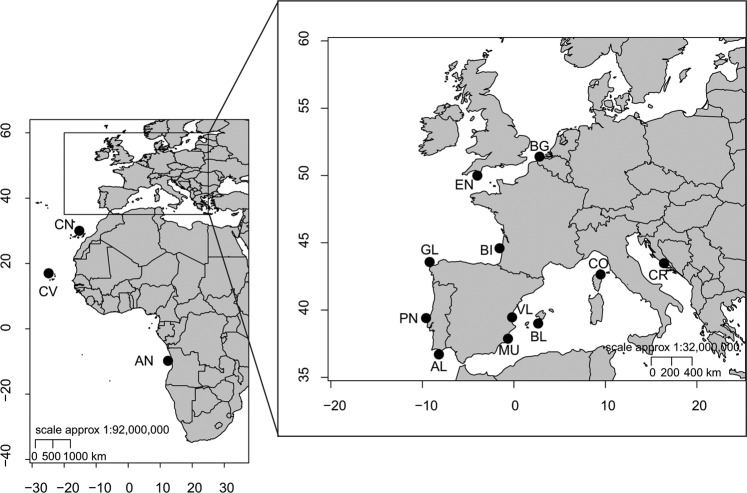
Sampling locations of *Spondyliosoma cantharus* in the Eastern Atlantic Ocean and the Mediterranean Sea. The position of samples with black dots is only indicative. Acronyms for locations as in sampling section.

A total of 263 black seabream samples were collected from 9 locations along the Eastern Atlantic and 5 locations in the Mediterranean Sea (Fig. 5, Supplementary Table S1). Samples were obtained from scientific cruises, carried by national fisheries institutes from Belgium, England, France, Spain and Angola, and purchased at the market in Portugal (Peniche and Algarve), Canary Island, Cape Verde, Corsica and Croatia. Individuals from English Channel (EN), Bay of Biscay (BI), Galicia (GL), Algarve (AL), Canary Island (CN) and Angola (AN) were frozen and sent to Lisbon. After arrival a piece of fin was removed and preserved in 96% ethanol. Samples from all the other locations, Belgium (BG), Peniche (PN), Cape Verde (CV), Murcia (MU), Valencia (VL), Balearic Islands (BL), Corsica (CO) and Croatia (CR), were taken from fresh fish and stored in 96% ethanol.

### DNA extraction, amplification, sequencing and alignment

Genomic DNA was extracted from fin samples with the REDExtract-N-Amp kit (Sigma-Aldrich) following the manufacturer’s instructions. Mitochondrial DNA cytochrome *b* (cyt*b*) and nuclear DNA first intron of the S7 ribosomal protein gene (S7) were amplified by polymerase chain reaction (PCR). For cyt*b*, amplification of the region of interest was obtained with pair of primers purposely designed by the authors, based on 9 aligned cyt*b* complete sequences of *Spondyliosoma cantharus* obtained from GenBank (http://www.ncbi.nlm.nih.gov/genbank) (accession numbers: DQ198007, EU036508/9, EF439602, EF439237, EF427607/8 and EU224083/4): BRBcytbF (forward) 5′-GCTGACTCATCCGAAATCTT-3′ and BRBcytbR (reverse) 5′-ATGTAGGGGTCTTCAACTGG-3′. S7 amplifications were performed with the following pair of primers: S7RPEX1F (forward) 5′-TGGCCTCTTCCTTGGCCGTC-3′ and S7RPEX2R (reverse) 5′-AACTCGTCTGGCTTTTCGCC-3′^39^. PCR amplification reactions were performed in a 20 μl total-reaction volume with 10 μl of REDExtract-N-ampl PCR reaction mix (Sigma–Aldrich), 0.8 μl of each primer (10 mM), 4.4 μl of bidistilled water and 4 μl of template DNA. An initial denaturation at 94 °C for 5/3 min was followed by 30 cycles (denaturation at 94 °C for 45 s, annealing at 56 °C for 30/60 s, and extension at 72 °C for 1 minute) and a final extension at 72 °C for 10 minutes on a BioRad Mycycler thermal cycler (values cyt*b*/S7, respectively). PCR products were purified with the SureClean kit (Bioline) following the manufacturer’s protocol, and the same primers were used for the sequencing reaction provided by Macrogen (http://www.macrogen.com, 716 samples) and STABVIDA (http://www.stabvida.net/, 108 samples).

Sequences were aligned using Clustal W^40,41^ in BIOEDIT^42^. S7 haplotypes of length-variant heterozygotes were determined using Mixed Sequence Reader^43^, and manual adjustments were made whenever necessary. Allelic states of S7 sequences were estimated using the Bayesian programme PHASE 2.1^44–46^. Five runs with different seeds for the random number generator and 500 iterations as burn-in, 500 main iterations and a thinning interval = 1 were performed to check for consistency across results. All runs returned consistent allele identities.

### Population structure

The relationship between haplotypes was analysed by haplotype networks built with the software PopART^47^ using a TCS network^48^. For cyt*b* network some additional GenBank sequences from Madeira (MD) and Greece (GR) were added to extend the sampling area (GenBank accession nos. EF439237, EU036508/9).

Hierarchical analysis of molecular variance (AMOVA)^49^ was used to estimate genetic structure among and between regions. Estimates of genetic divergence among regions and among populations within region were calculated with the fixation index F_ST_ and Φ_ST_^49^ as implemented in ARLEQUIN V3.5^50^, with p-values being corrected for multi comparisons by Benjamini-Hochberg (BH) false discovery rate method^51^.

The correlation between geographical distance, measured along the coastline, and F_ST_ was computed with the Mantel test^52,53^, also in ARLEQUIN with 1 × 10^5^ permutations.

### Diversity analysis

Diversity indices (number of haplotypes, % of private haplotypes, haplotype and nucleotide diversities, mean pairwise differences and number of polymorphic loci)^54,55^ were estimated for all populations using ARLEQUIN V3.5^50^. Graphical presentation of diversity indices was obtained using ggplot2 package^56^ for R^57^.

### Demographic analysis

Demographic history for the region structure depicted for cyt*b* by the haplotype networks and AMOVA analyses was investigated in ARLEQUIN V3.5 with neutrality tests, Tajima’s D^58^ and Fu’s F_S_^59^, the R_2_^14^ was also estimated using package pegas^60^ for R^57^. Mismatch distribution analysis was also performed for each region and compared to the distribution expected in populations affected by sudden expansion (1 × 10^5^ replicates), under the assumption of selective neutrality, in which a unimodal distribution is expected^61^. The sum of squared deviation (SSD) and raggedness index (Hri) were used to detect departure between observed and expected distributions. If evidence of expansion was found (p values > 0.05) the *τ* parameter of demographic and spatial expansions was used to estimate the time since the expansion (t, in years), using the equation t = (*τ* × n)/(2 × *μ* × *k*), where *μ* is the nucleotide mutation rate, *k* is the sequenced number of nucleotides and n is the generation time (equal to age for sexual maturity)^62^. Since there is no information on cyt*b* mutation rate for *S. cantharus*, the values used in the present study were 2% per nucleotide per Myr based on^16^. The generation time used was 3.8 years, according to^63^.

Past population demography of *S. cantharus* was also inferred using Bayesian skyline plot (BSP)^64^, employing the Bayesian MCMC coalescent method and a strict clock as implemented in BEAST 1.8.4^65^. Best-fit model of nucleotide substitution for cyt*b* was estimated with ModelFinder in IQ-TREE^66^. The TN + G4 was the model selected and an average mutation rate of 2% per nucleotide per Myr was used. The Bayesian distribution was generated with 1 × 10^8^ Markov Chain Monte Carlo (MCMC) steps, and the convergence of parameters was visually checked with effective samples sizes (ESS) of estimates over 200 with TRACER 1.7.1^67^. The time to most recent common ancestor (tMRCA) and the median and corresponding credibility intervals of the BSP were assessed for each population.

## Supplementary information

Supplementary information.

## Data Availability

The sequences generated under this study are available from the GenBank database and accession numbers are given in Results section.
