# Supplementary material for: Highly regional population structure of Spondyliosoma cantharus depicted by nuclear and mitochondrial DNA data
Source: Sci Rep. 2020 Mar 4;10:4063. doi: 10.1038/s41598-020-61050-x (PMC7055218; doi:10.1038/s41598-020-61050-x)
Supplement: Supplementary file 1 — Supplementary information. [file 41598_2020_61050_MOESM1_ESM.pdf]

Highly regional population structure of *Spondyliosoma cantharus* depicted by nuclear and mitochondrial DNA data

Ana Neves[1,2,*], Ana Rita Vieira[1,2], Vera Sequeira[1,2], Rafaela Barros Paiva[2], Leonel Serrano Gordo[1,2], Octávio S. Paulo[1,3]

1 Departamento de Biologia Animal, Faculdade de Ciências, Universidade de Lisboa, Campo Grande, 1749-016 Lisboa, Portugal

2 MARE – Marine and Environmental Sciences Centre, Faculdade de Ciências, Universidade de Lisboa, Campo Grande, 1749-016 Lisboa, Portugal

3 cE3c - Centre for Ecology, Evolution and Environmental Changes, Faculdade de Ciências, Universidade de Lisboa, Lisboa, Portugal

* Corresponding author: e-mail: amneves@fc.ul.pt

Table S1. Diversity measures for the sample areas and region groups of *Spondyliosoma cantharus* for mtDNA cyt*b* and nDNA S7. Number of sequences (N), number of haplotypes (Nh), private haplotypes (Hp), haplotype diversity (Hd), nucleotide diversity (π) and mean number of pairwise differences (PD). NEAT – North Eastern Atlantic; WAFT – West African Transition; GLGN – Gulf of Guinea; MEDS – Mediterranean Sea. Acronyms for populations as in Sampling section

	**cytb**						**S7**					
Area	N	Nh	Hp	Hd	π	PD	N	Nh	Hp	Hd	π	PD
**NEAT**	**137**	**26**	**24 (92%)**	**0.768**	**0.002**	**1.525**	**170**	**52**	**48 (92%)**	**0.848**	**0.005**	**2.443**
BG	3	3	0 (0%)	1.000	0.003	2.000	4	4	2 (50%)	1.000	0.008	4.000
EN	22	7	3 (43%)	0.671	0.002	1.134	10	9	7 (78%)	0.978	0.010	4.800
BI	21	7	1 (14%)	0.771	0.002	1.324	24	9	4 (44%)	0.706	0.003	1.565
GL	21	6	1 (16%)	0.723	0.002	1.314	10	6	2 (33%)	0.778	0.004	1.956
PN	24	10	5 (50%)	0.855	0.003	1.931	42	21	9 (43%)	0.905	0.005	2.541
AL	21	10	4 (40%)	0.857	0.003	2.181	28	16	8 (50%)	0.907	0.006	2.817
CN	25	7	4 (57%)	0.697	0.002	1.307	52	13	7 (54%)	0.780	0.004	1.968
**WAFT**	**25**	**4**	**4 (100%)**	**0.410**	**0.001**	**0.440**	**42**	**7**	**7 (100%)**	**0.512**	**0.001**	**0.678**
CV	25	4	4 (100%)	0.410	0.001	0.440	42	7	7 (100%)	0.512	0.001	0.678
**GLGN**	**14**	**8**	**8 (100%)**	**0.769**	**0.005**	**3.000**	**-**	**-**	**-**	**-**	**-**	**-**
AN	14	8	8 (100%)	0.769	0.005	3.000	-	-	-	-	-	-
**MEDS**	**87**	**27**	**25 (93%)**	**0.800**	**0.007**	**4.917**	**154**	**30**	**26 (87%)**	**0.842**	**0.004**	**1.948**
MU	14	7	3 (43%)	0.824	0.016	10.033	28	8	2 (25%)	0.741	0.002	1.185
VL	16	8	2 (25%)	0.858	0.019	12.150	18	10	3 (30%)	0.889	0.004	2.033
BL	13	4	1 (25%)	0.679	0.001	0.846	26	10	0 (0%)	0.871	0.005	2.308
CO	20	7	5 (71%)	0.742	0.001	1.005	32	11	2 (18%)	0.839	0.004	1.970
CR	24	14	9 (64%)	0.884	0.003	1.732	50	17	7 (41%)	0.861	0.004	2.105

Table S2. Gene flow among collecting sites of *Spondyliosoma cantharus* represented by F_{ST} (below diagonal) and Φ_{ST} (above diagonal). Significant values of probability assessed by permutation test with 10 000 replicates are shown in bold. NEAT – North Eastern Atlantic; MEDS – Mediterranean Sea. Acronyms for populations as in Sampling section.

	**Cytb**	NEAT								MEDS				
		EN	BI	GL	PN	AL	CN	CV	AN	MU	VL	BAL	CO	CR
NEAT	EN		-0.039	-0.034	-0.018	0.039	-0.028	**43.96**	**25.76**	**24.64**	**22.07**	**34.00**	**33.83**	**33.94**
	BI	-0.032		-0.05	-0.026	0.019	-0.031	**43.90**	**25.71**	**24.66**	**22.10**	**34.02**	**33.86**	**33.97**
	GL	-0.028	-0.039		-0.014	0.001.5	-0.034	**43.98**	**25.72**	**24.66**	**22.11**	**34.03**	**33.86**	**33.98**
	PN	-0.012	-0.017	-0.010		0.057	-0.004	**44.00**	**25.97**	**24.61**	**22.06**	**33.98**	**33.81**	**33.93**
	AL	0.024	0.011	0.000	0.027		0.003	**43.66**	**25.52**	**24.82**	**22.28**	**34.21**	**34.05**	**34.16**
	CN	-0.024	-0.024	-0.027	-0.002	0.004		**43.87**	**25.76**	**24.79**	**22.22**	**34.18**	**34.01**	**34.12**
	CV	**0.983**	**0.981**	**0.981**	**0.974**	**0.973**	**0.980**		**39.50**	**31.67**	**31.21**	**35.17**	**35.15**	**34.74**
	AN	**0.933**	**0.928**	**0.929**	**0.918**	**0.911**	**0.931**	**0.967**		**25.67**	**24.37**	**31.05**	**31.03**	**30.65**
MEDS	MU	**0.845**	**0.839**	**0.839**	**0.836**	**0.825**	**0.851**	**0.893**	**0.798**		-0.636	0.346	0.345	0.350
	VL	**0.795**	**0.788**	**0.788**	**0.788**	**0.776**	**0.803**	**0.864**	**0.755**	-0.061		0.824	0.810	0.819
	BAL	**0.971**	**0.967**	**0.9676**	**0956**	**0.953**	**0.967**	**0.984**	**0.940**	0.054	0.094		-0.030	-0.026
	CO	**0.969**	**0.967**	**0.967**	**0.957**	**0.955**	**0.967**	**0.981**	**0.945**	0.087	0.130	-0.035		0.015
	CR	**0.959**	**0.957**	**0.957**	**0.949**	**0.946**	**0.958**	**0.970**	**0.933**	0.092	0.137	-0.030	0.008	

	**S7**	NEAT							MEDS				
		EN	BI	GL	PN	AL	CN	CV	MU	VL	BAL	CO	CR
NEAT	EN		**0.142**	0.162	**0.198**	**0.156**	**0.151**	**7.575**	**0.322**	**0.350**	**0.423**	**0.493**	**0.485**
	BI	**0.088**		0.048	0.009.0	-0.004	0.011	**8.747**	**0.172**	**0.201**	**0.256**	**0.347**	**0.338**
	GL	0.046	0.034		0.014	0.049	0.042	**8.531**	**0.108**	0.117	**0.207**	**0.294**	**0.258**
	EN	**0.088**	0.000	-0.004		-0.021	0.021	**8.788**	**0.176**	**0.205**	**0.237**	**0.328**	**0.313**
	AL	**0.062**	-0.003	0.008	-0.007		0.011	**8.835**	**0.226**	0.259	**0.297**	**0.388**	**0.381**
	CN	**0.100**	0.003	0.021	0.010	0.008		**8.788**	**0.227**	**0.252**	**0.308**	**0.404**	**0.390**
	CV	**0.845**	**0.898**	**0.904**	**0.845**	**0.853**	**0.863**		**8.830**	**8.859**	**8.928**	**9.005**	**8.996**
	MU	**0.174**	**0.113**	**0.088**	**0.077**	**0.101**	**0.115**	**0.909**		0.010	-0.021	0.045	0.013
MEDS	VL	**0.122**	**0.104**	0.054	**0.076**	**0.091**	**0.113**	**0.892**	0.012		0.005	**0.120**	0.029
	BAL	**0.145**	**0.115**	**0.081**	**0.087**	**0.103**	**0.130**	**0.873**	-0.011	0.001		0.034	-0.028
	CO	**0.185**	**0.161**	**0.130**	**0.124**	**0.141**	**0.170**	**0.880**	0.026	**0.057**	0.017		0.002
	CR	**0.191**	**0.147**	**0.108**	**0.120**	**0.141**	**0.161**	**0.861**	0.003	0.013	-0.012	0.001	

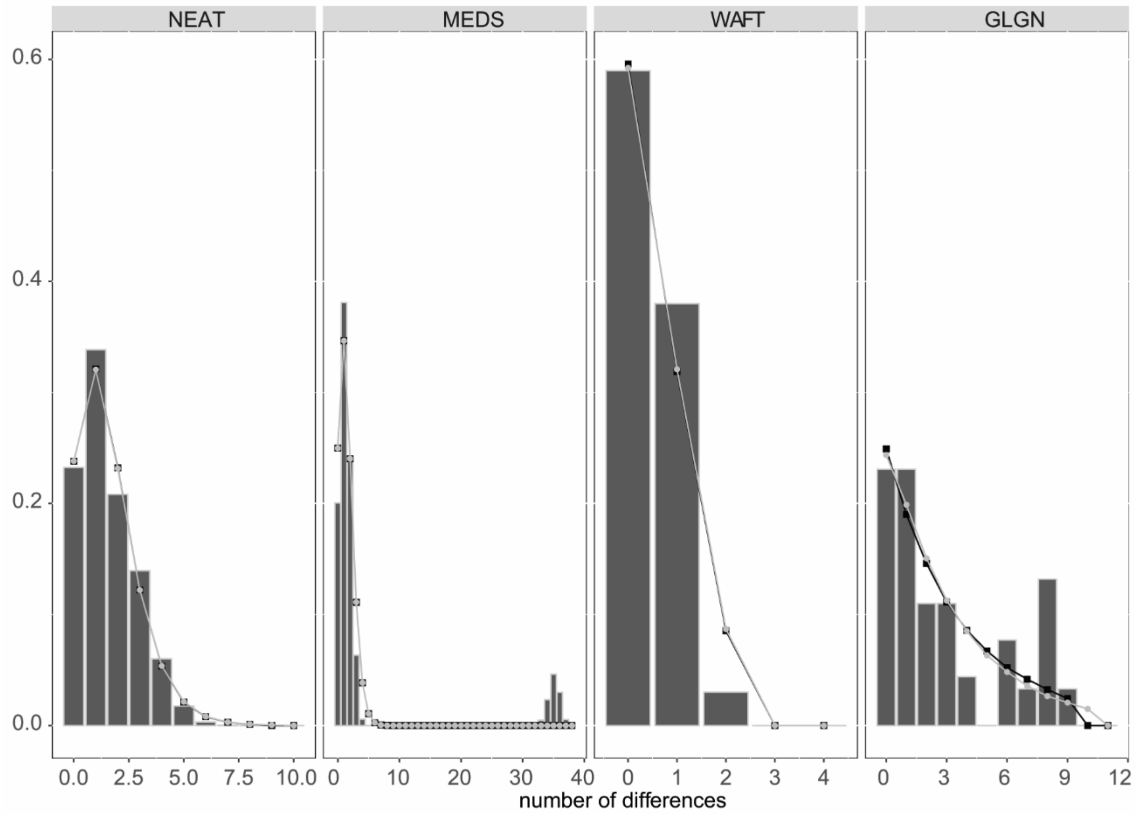

Fig. S1. Mismatch distributions for the mtDNA cyt*b* of *Spondyliosoma cantharus* in the four regions identified. NEAT – North East Atlantic; MEDS – Mediterranean Sea; WAFT – West African Transition; GLGN – Gulf of Guinea. Bars represent data observed, grey dots represent simulated spatial expansion and black squares represent simulated demographic expansion.
